# Motor function benefits of visual restoration measured in age-related cataract and simulated patients: Case-control and clinical experimental studies

**DOI:** 10.1038/srep14595

**Published:** 2015-09-30

**Authors:** Masahiko Ayaki, Takeo Nagura, Yoshiaki Toyama, Kazuno Negishi, Kazuo Tsubota

**Affiliations:** 1 Departments of Ophthalmology, Keio University School of Medicine, Tokyo, Japan; 2 Orthopedic surgery, Keio University School of Medicine, Tokyo, Japan

## Abstract

The aim of the present study was to measure gait velocity in cataract and simulated patients. The study was performed on 239 cataract patients, 115 age-matched subjects, and 11 simulated patients. We measured gait velocity and analyzed gait using a three-dimensional motion analysis system. Mean gait velocity before and 2 and 7 months after cataract surgery was 0.91 ± 0.19, 1.04 ± 0.21, and 1.06 ± 0.21 m/s, respectively, for males and 0.84 ± 0.22, 0.91 ± 0.24, and 0.92 ± 0.25 m/s, respectively, for females. The increase after surgery was significant in both groups at 7 months (*P* < 0.05). Gait velocity was significantly slower in cataract patients compared with controls before surgery, but no longer different after surgery. In simulated patients, mean velocity was 87.0 ± 11.4% of normal vision with a 3° visual field and 92.4 ± 12.3% of normal when counting fingers. Initial velocity was 89.1 ± 14.6% of normal vision with a 3° visual field and 92.7 ± 11.6% of normal when counting fingers. There was a significant difference between normal and impaired visual function (*P* < 0.05). The results demonstrate the close relationship between visual function and gait in cataract patients and simulated patients.

Age-related decline in motor function critically affects survival^1^,^2^,^3^,^4^,^5^,^6^. Healthy gait and posture are essential for the prevention of falls in older people, and they are controlled by the somatosensory, vestibular, and visual systems. Traditionally, neurology, otolaryngology, orthopedics, and rehabilitation medicine have been concerned with gait and posture, and ophthalmological evaluation of gait has been understudied even though motor function is directly associated with visual function^7^,^8^,^9^,^10^,^11^,^12^,^13^,^14^,^15^,^16^. A positive relationship between eye disease and motor function has been suggested in a number of cohort studies of patients with cataract^17^,^18^,^19^,^20^,^21^,^22^,^23^,^24^,^25^, macular degeneration^15^,^26^, or glaucoma^27^,^28^. The contribution of vision to mobility has also been examined in patients with simulated visual defects, such as closed eyes^29^ or wearing prism glasses^30^. Many of the previous studies into the link between cataract and mobility have been community-based epidemiological studies evaluating the risk of falls based on participants’ diaries and chart review. Two clinical studies have evaluated mobility in cataract patients. Schwartz *et al.*^21^ reported improved postural control for 23 cataract patients 1–4 months after surgery. In another study, Durmus *et al.*^23^ undertook comprehensive evaluation of motor function in 36 cataract patients before and 4 weeks after surgery and demonstrated improvements in gait analysis, as evaluated using the Biodex Stability System, time to “up and go”, the Tinetti assessment tool, and a functional reach test.

Cataract is a very common geriatric disease. As such, a clinical study into preoperative motor function decline and postoperative improvement in cataract patients could be of considerable benefit to public health. Ophthalmological interventions and experimental simulation of vision loss using eyewear could provide more useful information regarding the contribution of vision to motor function than conventional methods. In the present study we analyzed 239 cataract patients to explore correlations between gait velocity and ocular and systemic parameters. Gait velocity has been proposed as a relevant clinical indicator of well being in older adults and has been shown to predict survival^1^. Unlike fall frequency, gait velocity can be evaluated objectively in the eye clinic. All patients were examined and followed-up by board-certified ophthalmologists, certified orthoptists, and registered nurses throughout the study period. We also measured the gait velocity of subjects with normal vision in the same setting as cataract patients to compare gait velocity between the two groups. Finally, standardized technology for motion analysis (i.e. the point cluster method using a three-dimensional motion capture system) was used to evaluate gait velocity in subjects with simulated vision impairment (“simulated” patients) to determine whether the change in visual function, as a single factor, affected motor function.

## Methods

### Study institutions and Institutional Review Board approval

The Institutional Review Board and Ethics Committee of Keio University School of Medicine approved this study and the methods were carried out in accordance with the approved guidelines. All cataract and simulated patients provided written informed consent before taking part in the study. Subjects were recruited to the study from cataract patients and individuals with normal vision visiting eye clinics at the National Hospital Organization Saitama Hospital (Wako, Japan) and International Health and Welfare University Mita Hospital (Tokyo, Japan) between May 2011 and April 2013. Simulated patients were recruited from Keio University Hospital (Tokyo, Japan) between September 2013 and February 2014.

### Gait velocity of cataract patients

The mean (±SD) age of the 239 cataract patients (145 women; 61%) was 74.5 ± 8.0 years. Patients were undergoing phacoemulsification and intra-ocular lens (IOL) implantation. Cataract patients were enrolled consecutively from the eye clinic and not biased. All surgeries were performed under topical anesthesia and a soft acrylic IOL (SN60WF [Alcon Laboratory, Fort Worth, TX, USA] n = 155; SA60AT [Alcon Laboratory] n = 84) was inserted by experienced surgeons. Best corrected visual acuity was converted to logarithm of minimal angle of resolution (LogMAR) units, and cataract opacity in the optical axis accounting for visual disturbance was classified as posterior subcapsular cataract, nuclear sclerosis (greater than Grade 2 on Emery–Little classification), or central cortical opacity. Some patients had mixed-type opacity. All patients completed the Japanese version^31^ of the National Eye Institute Visual Function Qestionnaire-25 (VFQ-25) before and 2 months after surgery. A trained technician measured 4-m gait velocity on a flat, straight track using a digital stopwatch. Patients were asked to walk at their preferred velocity from a standing position. This method has been used in many studies and meta-analyses^1^, and is considered appropriate for comparisons with the general population. Graders were not masked as to subject characteristics. Subjects who required a cane or walking aid and those with significant orthopedic and/or neurological complications were excluded from subsequent analysis.

### Gait velocity of age-matched controls with normal vision

The control group consisted of 115 subjects with normal vision visiting the eye clinic for non-vision-related problems, including an eye health check, vitreous floaters, and ocular discomfort. Inclusion criteria for the control group were age between 64 and 84 years, not using a cane or walking aid, and normal vision (best corrected visual acuity >20/25 in both eyes). Exclusion criteria were vision-affecting eye disease, diabetes, brain infarction, and orthopedic and/or neurological complications, including paralysis, Parkinson’s disease, and a history of orthopedic surgery. Subjects were asked to walk at their preferred velocity in the examination room in the eye clinic, and 4-m gait velocity was measured once by a trained technician using a digital stopwatch.

### Motion analysis of simulated patients

**Figure 1:**
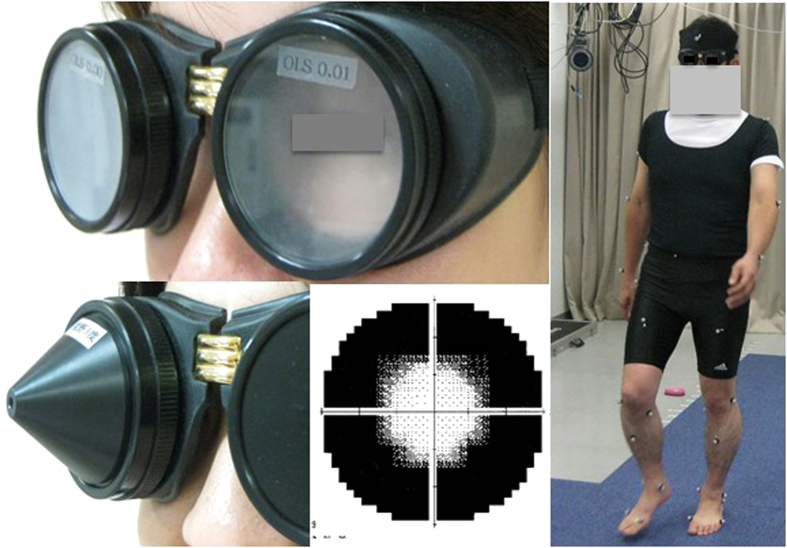
Simulation goggles and simulated visual field. “Simulated patients” wore goggles (top left) to simulate reduced visual function to the vision of 20/600. Representative results of visual field analysis (Humphrey Field Analyzer 30-2 program; Carl Zeiss, Jena, Germany) with goggles restricting the visual field to 3° show a successful simulation effect (bottom left and center). Motion analyses were performed using a motion capture system comprising eight cameras and a force plate (right).

Eleven healthy male volunteers (aged 30–60 years; mean age 45 years; best corrected visual acuity 20/15) were recruited as simulated patients. Gait was evaluated in these subjects at the Gait Laboratory of the Department of Orthopedic Surgery using skin markers attached over the sacrum as part of a three-dimensional motion capture system (Qualysis^®^; Qualysis, Gothenburg, Sweden)^32^,^33^ with eight cameras synchronized to a force plate (frequency 600 Hz; Type 4060-10; Bertec, Columbus, OH, USA). This point cluster method enables measurement of velocity, vector, acceleration, step length, pitch, and many other parameters of motor function. Gait velocity was analyzed using the markers on the sacrum, which can best trace the motion of the center of gravity in a subject. The data sampling frequency was 120 Hz, and mean and initial (mean velocity during the first 0.1 s of the first step) velocities were calculated. Simulated patients wore goggles with a smoked surface or pin hole (M. Takata Optical, Tokyo, Japan) to reduce visual function to a visual acuity of counting fingers and 20/600, or to reduce the visual field to 3°, respectively (Fig. 1). The simulated effects were confirmed in 10 healthy volunteers with normal vision (>20/15). The goggles simulated visual acuity within an error range of 10% for counting fingers at 20 cm and 20/600. The visual field was evaluated in simulated patients wearing goggles with a Humphrey Field Analyzer (Carl Zeiss, Jena, Germany; 30-2 program) and the mean deviation was −29.9 ± 1.9 dB. The real visual field was ~3° (Fig. 1). This simulation is apparently equivalent to the visual field loss in advanced glaucoma; however, it is not very accurate and not a validated method for simulating advanced glaucomatous vision loss^34^.

Simulated patients were asked to walk 6 m on a flat, straight track at their preferred velocity from a standing position. The floor was solid and without obstacles in order to evaluate gait under various simulated visual impairments in a simple environment. To exclude carry-over effects, participants were divided into two groups; the first group of six was examined under normal vision conditions first and then when wearing the goggles; the second group of five was examined in the reverse order. Based on preliminary experiments, we used three goggles simulating the lowest visual function to achieve significant differences and to avoid learning effects by using too many goggles.

### Statistical analysis

Where appropriate, data are given as the mean ± SD. Data were analyzed using ANOVA and *t-*tests. Correlations were evaluated using Pearson’s product–moment correlation. All analyses were performed using StatFlex (Atech, Osaka, Japan) and SPSS version 21 (SPSS Inc., Chicago, IL, USA). *P* < 0.05 was considered significant.

## Results

**Figure 2:**
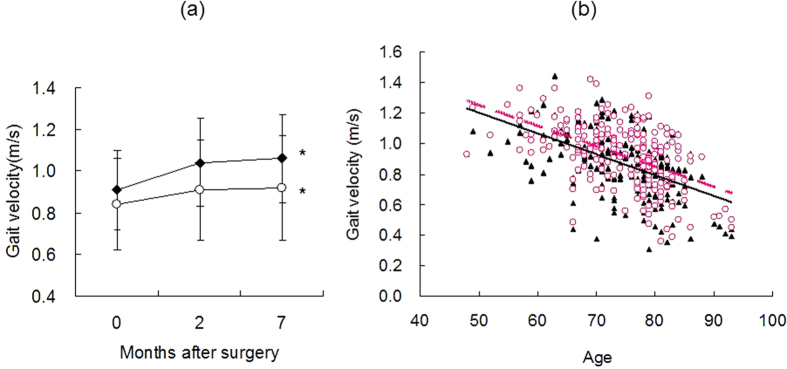
(**a**) Four-meter gait velocity in male (closed symbols) and female (open symbols) cataract patients before and 2 and 7 months after surgery. Gait velocity increased continuously up to 7 months after surgery with statistical significance in both groups (*P < 0.05 vs preoperative velocity, paired *t-*test). (**b**) Scatter plot of gait velocity versus age of cataract patients before (closed symbols) and 2 months after (open symbols) surgery. Note that age was strongly correlated with gait velocity. The regression lines were *y* = −0.0136*x* + 1.8845; *R*^2^ = 0.2753 (solid line) for before surgery and *y* = −0.0134*x* + 1.9175; *R*^2^ = 0.2743 (dashed line) for after surgery.

Demographics and gait velocity for cataract patients are given in Table 1. Generally, gait velocity was significantly greater for male than female patients before and after surgery. Mean gait velocity increased significantly up to 7 months after surgery in both groups (Fig. 2; *P* < 0.05, paired *t-*test). Mean gait velocity in male (n = 40; mean age 74.8 ± 5.6 years) and female (n = 75; mean age 75.0 ± 6.7 years) controls with normal vision was 0.92 ± 0.21 and 0.95 ± 0.17 m/s, respectively. The difference in gait velocity between cataract patients and controls was significant before surgery (*P* < 0.001, unpaired *t-*test and Mann–Whitney *U*-test), but not 2 months after surgery, in both gender groups.

Stepwise multiple regression analysis of gait velocity and related parameters revealed that preoperative velocity was significantly correlated with sex (*P* < 0.01, Pearson’s product moment correlation), age (*P* < 0.001), height (*P* < 0.001), visual acuity in the worse eye (*P* < 0.05), and VFQ-25 score (*P* < 0.001) adjusted for age and sex (Table 2). The change in velocity 2 months after surgery was significantly correlated with the change in visual acuity in the worse eye (*P* < 0.05) and the presence of posterior subcapsular cataract adjusted for age and sex (*P* < 0.01). Graphic representation revealed a strong correlation between age and gait velocity (Fig. 2). Regression lines for before (*y* = −0.0136*x* + 1.8845; *R*^2^ = 0.2753) and 2 months after (*y* = −0.0134*x* + 1.9175; *R*^2^ = 0.2743) surgery demonstrate an increase in gait speed after surgery.

**Figure 3:**
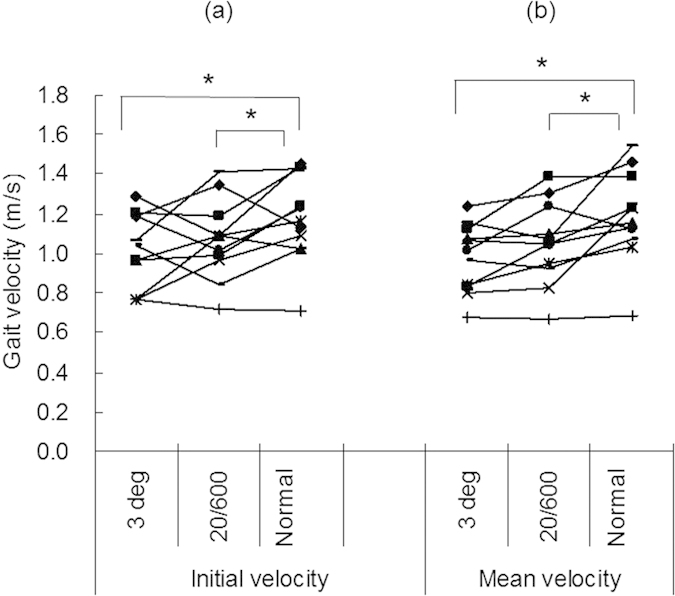
(**a**) Initial velocity (mean velocity during the first 0.1 s of the first step) and (**b**) mean velocity in subjects in whom low visual function was simulated by reducing visual acuity to 20/600 and counting fingers, and reducing the visual field to 3°. Note that gait velocity was correlated with visual function. There was a significant difference between values obtained under normal vision conditions (visual acuity > 20/15) and each simulated low-vision condition (*P < 0.05, paired *t-*test).

Simulated patients exhibited significantly reduced gait velocity when visual function was impaired compared with normal-vision conditions (Fig. 3). Mean velocity under simulated low-vision conditions was 86.0 ± 15.1% of normal (*P* <0.05 vs normal vision, paired *t*-test) with a visual acuity of 20/600, 81.8 ± 23.6% (*P* < 0.05) when counting fingers, and 79.8 ± 15.4% (*P* < 0.01) with a visual field of 3° (*P* < 0.01). Initial velocity in simulated patients was 90.5 ± 8.8% of normal (*P* < 0.05) with a visual acuity of 20/600, 82.5 ± 13.8% (*P* < 0.05) when counting fingers, and 84.3 ± 9.7% (*P* < 0.05) with a visual field of 3° (*P* < 0.05).

## Discussion

The results of the present study demonstrate that gait velocity increases 2 months after cataract surgery and further increases at 7 months, despite the fact that these elderly patients are also 7 months older. To the best of our knowledge, the present study is the largest clinical case series of gait velocity in patients undergoing cataract surgery. The improvement in gait velocity was not dependent on age, sex, or height, indicating that sensory, neural, or psychological robustness may be more important than musculoskeletal components for achieving and maintaining a healthy gait in older people. Therefore, age-related declines in gait may be due not only to physical frailty as a consequence of aging, but also to a fear of falling as a result of low visual recognition exacerbated by eye disease. It was interesting that, prior to surgery, the cataract patients in the present study had significantly slower gait velocity than age-matched subjects with normal vision without cataract but, after surgery, cataract patients walked faster than the control subjects even through some of them had diabetes and other comorbidities that could possibly suppress gait. Our clinical impression was that the cataract patients decided to undergo surgery only when they were mentally and physically robust enough to tolerate the procedure. According to recent reports, postoperative improvements in sleep quality or mental status are no longer evident in some cases 1 year after surgery^35^,^36^,^37^, and we speculate that the sustainability of non-physical improvements after cataract surgery may depend on individual patients. Nevertheless, improvements in gait are supported, at the very least, by sustained restoration of the visual sensory system, and so longer effects may be expected compared with improvements in mental status.

Most previous studies of gait speed were performed as part of a health check for a large population, so very simple methods (in terms of efficacy and safety) were used to determine gait speed, such as measurements made over a short distance using a stopwatch. In the present study, we chose to measure walking velocity over a distance of 4 m because the reproducibility and standardization of this technique have been established in many studies^1^, even when measured by different graders. It is very important to evaluate another control group of cataract patients who did not undergo surgery, to determine if repeat testing results in improvements in gait velocity.

In the present study, visual acuity in the worse eye was correlated with preoperative gait velocity and postoperative improvement, which accords with the study of Matsuo *et al.*^30^, who found a close relationship between binocular function and postural stability. Thus, binocular vision may be more important for gait than good monocular vision. Vision and mobility studies also suggest contrast sensitivity^21^, visual field^27^, illumination^16^, and the use of sedatives^10^ are significantly correlated with postural stability. Geriatricians and eye care providers are advised to take care of visual function in older people to enable them to walk safely.

With regard to the type of cataract opacity, patients with posterior subcapsular cataract exhibited a greater increase in gait velocity compared with patients with other types of opacities. These findings support those of previous studies that posterior subcapsular cataract is a significant prognostic determinant of cataract surgery for quality of life and sleep^31^,^37^. This type of cataract has a greater adverse effect on vision under conditions of bright ambient lighting. Visual acuity is usually tested in an indoor examination room, using black letters on a white background. Patients with posterior subcapsular cataract usually have much worse acuity in a brightly lit environment than in a darker examination room. Based on the clinical experiences of eye surgeons, we speculate that there is response shift phenomenon^38^ whereby patients with posterior subcapsular cataract do not recognize their preoperative disability until their visual function recovers after surgery. Consequently, patients with posterior subcapsular cataract may enjoy the best outcomes after surgery, even though preoperative parameters do not differ among the different types of cataract opacities.

Three-dimensional motion capture is a modern technology for analyzing motion under normal and pathological conditions. It is commonly used in orthopedic evaluation by capturing the motion of each joint and bone^32^,^33^. We attempted to exclude the possible bias of age and other systemic conditions on gait velocity by simulating low-vision conditions. The results from simulated patients demonstrated that motor function decreased significantly with visual impairment alone and confirmed that visual function plays an important role in postural control. Visual restoration may potentially contribute to longer survival since recent epidemiological studies indicating that the mortality rate is lower in postoperative cataract patients than in those without surgery^39^,^40^. Cataract surgery is also suggested to be a potential anti-aging therapy in terms of vision, mobility^17^,^18^,^19^,^20^,^21^,^22^,^23^,^24^,^25^, cognitive function^41^,^42^, and sleep^24^,^25^,^36^,^37^,^43^,^44^. Further investigations should be performed to evaluate neural and musculoskeletal improvements after cataract surgery that could sustain improvements in gait velocity.

The limitations of the present study include the type of control subjects for old cataract patients, simulated patients, and the learning curve of participants. There are many systemic conditions that can potentially affect gait velocity and most older adults may have age-related disabilities and comorbidities. Physicians and nurses observed the gait and sitting and standing posture of subjects with normal vision to confirm that they were eligible to be enrolled in the study as normal controls. It was not ethical to ask older participants to walk while wearing simulation goggles because of safety concerns. However, we believe the healthy simulated patients in the present study successfully served as a relevant experimental model and contributed to reasonable results. With regard to the possible learning curve of participants, gait velocity measurements were performed only once using a very simple method that required no skill or practice. Measurements after surgery were performed 2 and 7 months after the initial measurement and, after this time, the patients may not remember how they walked in the initial assessment. Although graders were not masked as to subject characteristics, we do not believe this resulted in bias because the graders used the same simple method for all subjects.

In conclusion, we found that the gait velocity of cataract patients increased over 7 months after surgery and exceeded that of age-matched controls with normal vision. Three-dimensional motion analysis of simulated patients also demonstrated a significant correlation between visual function and gait velocity. The results of the present study confirm motor function benefits after visual restoration by cataract surgery.

## Additional Information

**How to cite this article**: Ayaki, M. *et al.* Motor function benefits of visual restoration measured in age-related cataract and simulated patients: Case-control and clinical experimental studies. *Sci. Rep.* **5**, 14595; doi: 10.1038/srep14595 (2015).

## Figures and Tables

**Table 1:** Demographics and gait velocity in cataract patients.

	All patients	Male	Female	*P*-value
Gait velocity (m/s)				
Before surgery	0.87 ± 0.21	0.91 ± 0.19	0.84 ± 0.22	0.01**
2 months after surgery	0.96 ± 0.23	1.04 ± 0.21	0.91 ± 0.24	0.04**
7 months after surgery	0.98 ± 0.24*	1.06 ± 0.21*	0.92 ± 0.25*	0.04**
Model 1: systemic parameters				
No. patients	239	95	144	
Age (years)	74.4 ± 8.0	72.1 ± 8.8	76.0 ± 7.1	<0.001**
Height (cm)	155 ± 9	163 ± 6	149 ± 6	<0.001**
Weight (kg)	56.2 ± 11.4	63.4 ± 11.0	51.3 ± 8.8	<0.001**
BMI (kg/m^2^)	23.3 ± 3.5	23.7 ± 3.6	22.9 ± 3.4	0.107
Diabetes (%)	23.8	23.7	22.9	0.310
SBP (mmHg)	131.3 ± 18.1	129.8 ± 18.7	132.4 ± 17.6	0.326
Model 2: vision-related parameters				
VFQ-25 score				
Before surgery	64.3 ± 16.4	68.4 ± 13.8	61.4 ± 17.6	<0.001**
After surgery	79.8 ± 12.5*	80.6 ± 10.8*	79.4 ± 13.6*	0.477
Visual acuity before surgery				
In the better eye	0.16 ± 0.29	0.11 ± 0.19	0.20 ± 0.34	0.01**
In the worse eye	0.63 ± 0.80	0.58 ± 0.68	0.67 ± 0.87	0.390
Visual acuity 2 months after surgery				
In the better eye	−0.03 ± 0.09*	−0.05 ± 0.06*	−0.02 ± 0.10*	0.003**
In the worse eye	0.11 ± 0.46*	0.09 ± 0.31*	0.12 ± 0.53*	0.579
% Hyperopic (either eye ≥ +3·00^D^)	7.1	7.4	6.9	0.900
% Myopic (either eye ≤ −3·00^D^)	25.5	31.6	21.5	0.081
Model 3: type of cataract opacity				
Posterior subcapsular	38.5%	49.5%	31.3%	0.012**
Nuclear (>Grade 2)	23.4%	24.7%	22.4%	0.721
Central cortical	47.5%	42.3%	51.0%	0.193

Data are given as the mean ± SD or as the percentage of patients in each group, as appropriate. *P < 0.05 compared with before surgery (paired *t-*test); **P < 0.05 for male versus female (unpaired *t-*test).

BMI, body mass index; SBP, systolic blood pressure; LogMAR, logarithm of the minimum angle of resolution; VFQ-25, visual function questionnaire 25.

**Table 2:** Stepwise multiple regression analysis of gait velocity and related parameters.

	Preoperative gait velocity		∆Gait velocity^A^	
	β	*P*-value	Β	*P*-value
Model 1: vision-related parameters				
LogMAR in the better eye	−0.11	0.06	0.02	0.79
LogMAR in the worse eye	−0.12	0.04*	0.07	0.29
∆LogMAR in the worse eye	—	—	0.55	0.04*
Myopia > −3.0D in either eye	0.02	0.70	0.08	0.27
Preoperative VFQ-25 score	0.28	0.00*	−0.05	0.46
∆VFQ-25 score	—	—	0.04	0.55
Model 2: cataract opacity				
Nuclear sclerosis	−0.09	0.12	0.02	0.74
Posterior subcapsular	−0.07	0.30	0.20	0.01*
Central cortical	0.09	0.19	0.02	0.81
Model 3: systemic parameters				
Age	−0.52	0.00*	−0.06	0.50
Sex	0.19	0.01*	−0.02	0.72
Height	0.39	0.00*	−0.12	0.32
BMI	−0.05	0.41	−0.12	0.11
Diabetes	−0.03	0.68	0.02	0.81
SBP	−0.07	0.31	0.11	0.14

*P < 0.05, Pearson’s product–moment correlation.

Male = 1, female = 0, diabetic = 1, non-diabetic = 0.

^A^Changes in values (Δ) were calculated by subtracting preoperative values from values obtained 2 months after surgery. All values were adjusted for age and sex.

LogMAR, logarithm of the minimum angle of resolution; VFQ-25, visual function questionnaire 25; BMI, body mass index; SBP, systolic blood pressure.
